# Organizational Citizenship Behavior of Cabin Crew: A Taiwanese Case Study in a Post-Pandemic Context

**DOI:** 10.3390/bs16030449

**Published:** 2026-03-18

**Authors:** Kai-Chieh Hu, Yi-Ting Ruan

**Affiliations:** Department of Business Administration, Soochow University, Taipei City 100006, Taiwan

**Keywords:** organizational citizenship behavior, perceived organizational support, job crafting, organizational commitment, job burnout, cabin crew

## Abstract

The COVID-19 outbreak profoundly disrupted the aviation industry and reshaped cabin crew work conditions, increasing psychological strain and altering job resources. Against this backdrop, this study investigates the antecedents of organizational citizenship behavior among cabin crew members in a Taiwanese airline, focusing on job crafting, perceived organizational support, job burnout, and organizational commitment. A purposive and quota sampling method was employed to collect data through an online questionnaire from a Taiwanese airline company. The collected data was analyzed using structural equation modeling to verify the proposed model. The study found that job burnout does not significantly affect organizational citizenship behavior or organizational commitment and that job crafting does not significantly affect job burnout. In contrast, job crafting and perceived organizational support have a positive effect on organizational commitment and organizational citizenship behavior, whereas perceived organizational support has a negative effect on job burnout. Finally, the study discusses managerial implications and suggests directions for future research.

## 1. Introduction

In the airline passenger services industry, successful flight mission execution relies heavily on mutual support and organizational citizenship behavior (OCB) among cabin crew members. OCB involves voluntary actions beyond formal job requirements, which are essential for smooth operations (Organ, 1988; Fu, 2013). The COVID-19 pandemic thrust cabin crew into the forefront, requiring them to navigate strict quarantine protocols, safeguard public health, and maintain service standards (Alshaabani et al., 2021), while triggering widespread flight cancelations, income reductions, and operational restrictions that profoundly impacted organizational performance and employee behavior (Alshaabani et al., 2021). Consequently, the unprecedented challenges presented by the pandemic necessitated a deeper understanding of factors influencing discretionary behaviors, such as organizational citizenship behavior, especially among frontline aviation personnel (Long et al., 2022).

The airline passenger service sector provides a particularly suitable context for this investigation because cabin crew are frontline employees who must simultaneously ensure safety, comply with stringent health and security regulations, and deliver high-quality service during each flight mission. Cabin crew perform intensive emotional labor and are especially vulnerable to burnout, as they continuously manage passenger emotions and demands in a confined environment with irregular schedules. The COVID-19 pandemic further amplified these pressures by adding strict quarantine protocols, fear of infection, and operational disruptions, making cabin crew an ideal group for theorizing how job crafting and perceived organizational support shape burnout, commitment, and OCB under crisis and post-crisis conditions.

OCB refers to voluntary, self-initiated efforts by employees that exceed formal job requirements, thereby enhancing organizational effectiveness, improving work quality and efficiency, and contributing to organizational development (Casu et al., 2021; Organ, 1988; Farh et al., 1997). During the COVID-19 pandemic, cabin crew members were required to comply with stringent health protocols while maintaining their regular duties, making OCB particularly vital for sustaining airline operations (Alshaabani et al., 2021). Consequently, the capacity of cabin crew to engage in OCB amid these challenges has become a key focus for the industry.

Organizational commitment, defined as a psychological attachment to the organization that develops through compliance, identification, and internalization (Organ, 1988; O’Reilly & Chatman, 1986; Podsakoff et al., 2000), influences employees’ alignment with organizational goals, motivating them to maximize organizational benefits and engage in OCB. For cabin crew members, stronger organizational commitment fosters proactive OCB, such as assisting colleagues or voluntarily assuming additional responsibilities to ensure flight mission success (Fu, 2013). This is particularly critical during stressful times like the COVID-19 pandemic, when crew members must perform duties amid health risks and quarantine restrictions (Alshaabani et al., 2021). High organizational commitment enables crew members to exert extra effort, thereby sustaining high service standards under challenging conditions.

On the other hand, job burnout can negatively impact OCB (Larsen et al., 2012). Burnout, characterized by emotional exhaustion and cynicism, often arises in roles requiring intensive interpersonal interactions (Maslach et al., 1996; Maslach & Jackson, 1981). Cabin crew members, who engage in high levels of emotional labor, are particularly vulnerable to burnout (Richa et al., 2016). During the COVID-19 pandemic, factors such as strict epidemic prevention measures, fear of infection, and increased workloads exacerbated burnout by reducing crew autonomy, heightening role conflicts, and introducing role ambiguity. Understanding the relationships among burnout, organizational commitment, and OCB is crucial for exploring how these factors interact during crises.

Previous studies indicate that job crafting can enhance employee engagement, service recovery performance, and reduce turnover intentions among cabin crew (Karatepe & Eslamlou, 2017; Niessen et al., 2016). Wrzesniewski and Dutton (2001) define job crafting as proactive adjustments employees make to their tasks, relationships, or cognitions to achieve greater meaning and satisfaction in their work. As a proactive behavioral strategy, job crafting enables employees to better align their jobs with personal and organizational goals, thereby positively impacting various organizational outcomes (Niessen et al., 2016). For cabin crew members, receiving managerial support—such as from cabin supervisors or pursers—can boost confidence, focus, engagement, and performance through job crafting (Karatepe & Eslamlou, 2017). However, in the post-pandemic era, external constraints including limited shift changes, restricted rest time, and the requirement to wear full protective gear have hampered service delivery and the ability to adapt to service contexts and passenger needs. These challenges underscore the importance of internal job crafting among cabin crew. This study aims to examine whether the effects of job crafting on organizational commitment and job burnout align with prior research, particularly considering pandemic-related constraints.

Perceived organizational support (POS) is another key factor influencing organizational commitment and burnout. POS refers to employees’ perceptions of the extent to which their organization values their contributions and cares about their well-being (Eisenberger et al., 1986). For cabin crew members in emotionally demanding roles, POS serves as an essential resource for emotional recovery (Ji et al., 2019). During the COVID-19 pandemic, such support—exemplified by the provision of adequate protective gear—proved critical in helping crew members endure organizational challenges (Alshaabani et al., 2021). In turn, crew members perceiving high POS are more likely to reciprocate with elevated organizational commitment and OCB (Eisenberger et al., 1986; Gupta, 2022). Building on the foundational definition of POS by Eisenberger et al. (1986), this study integrates more recent evidence on POS in high-stress service and aviation settings during COVID-19 to ground its hypotheses in the current economic and organizational context.

While previous research on cabin crew has primarily focused on service quality and fatigue-related safety concerns (Gibbs et al., 2017; Mojaveri et al., 2019; Nesthus et al., 2007), there has been limited exploration of OCB and its underlying factors, particularly amid the COVID-19 pandemic, which intensified physical and psychological pressures on crew members (Alshaabani et al., 2021). These pressures suggest that POS (Eisenberger et al., 1986) and organizational commitment (Organ, 1988; Fu, 2013) may play a more prominent role in job performance, especially given the dynamic nature of cabin crew teams, where varying compositions per flight mission influence daily responsibilities, engagement, and burnout. However, prior studies have not fully examined the mediating roles of organizational commitment and burnout in the relationships involving job crafting (Karatepe & Eslamlou, 2017), POS, and OCB under pandemic conditions. This study addresses these gaps by investigating the interplay among job crafting, POS, organizational commitment, and burnout to develop a comprehensive theoretical framework for cabin crew OCB. By focusing on cabin crew in this context, the study contributes to extending theories of job crafting, perceived organizational support, burnout, and OCB to a high-stakes, team-based, and safety-critical service occupation where daily crew composition varies across flights, creating unique demands on discretionary behaviors and resource utilization.

In summary, this research examines the combined effects of job crafting and POS on organizational commitment and burnout during and after the COVID-19 pandemic, a period marked by aviation industry cost-cutting measures such as recruitment freezes, route suspensions, and furloughs that heightened employee anxiety (Alshaabani et al., 2021). It explores whether these effects persist for Taiwan’s cabin crew (Fu, 2013), analyzing the mediating roles of organizational commitment and burnout in influencing OCB, and offers actionable recommendations for airlines to sustain and enhance crew OCB in the post-pandemic era.

## 2. Literature Review

### 2.1. Organizational Citizenship Behavior

Organizational citizenship behavior refers to discretionary actions that extend beyond formal job requirements and are not explicitly prescribed or rewarded by official compensation systems (Aguiar-Quintana et al., 2020; Bennis et al., 1966; Organ, 1988). As Bateman and Organ (1983) noted, these behaviors are often taken for granted and not directly linked to task-specific duties, such as helping maintain a clean work environment or minimizing interpersonal conflicts. Organ (1988)’s definition—discretionary behaviors not outlined in job descriptions but contributing to organizational effectiveness—is the most widely accepted.

Although OCB is traditionally viewed as extra-role behavior, subsequent research distinguishes it into in-role behaviors (proactive actions exceeding formal responsibilities within designated roles (K. Lee & Allen, 2002; Podsakoff et al., 1997)) and extra-role behaviors (actions beyond assigned roles that enhance organizational reputation, attract talent, and foster societal connections (Bolino et al., 2012; Morrison, 1994, 2011; Robinson & Morrison, 2000)). This study adopts in-role OCB as its core framework, prioritizing proactive contributions within roles.

Organ (1988) classified OCB into five dimensions: altruism, conscientiousness, sportsmanship, courtesy, and civic virtue. Williams and Anderson (1991) further categorized it into OCB directed toward the organization (e.g., protecting resources and reputation) and toward individuals (e.g., assisting colleagues (Fu, 2013)). Synthesizing prior literature, (Podsakoff et al., 2000) identified seven dimensions: helping behavior, sportsmanship, organizational loyalty, organizational compliance, individual initiative, civic virtue, and self-development (Kuncoro & Wibowo, 2019).

In the airline context, studies on cabin crew OCB highlight its drivers and outcomes. For instance, Kuncoro and Wibowo (2019) showed transactional leadership enhances service-oriented OCB via job satisfaction, while Noermijati et al. (2024) found OCB mediates the link between followership and team performance. Similarly, Fu (2013) demonstrated organizational commitment positively predicts cabin crew OCB, moderated by HR policies. Given this study’s focus on how perceived organizational support and job crafting influence cabin crew OCB, it emphasizes organization-directed behaviors, guided by the framework by Buil et al. (2018).

### 2.2. Organizational Commitment

The concept of organizational commitment was introduced by Becker (1960) as “side-bet theory,” rationalizing inconsistencies between actions and attitudes. Contemporary views emphasize psychological bonds connecting employees to their organization through identification with its goals and desire to remain a member. Organizational commitment is defined as a psychological attachment to the organization that shapes employees’ stay-or-leave decisions and their willingness to exert effort for organizational goals (Cao et al., 2019; Meyer & Allen, 1997).

Porter et al. (1974) delineated three dimensions—attitudinal commitment, behavioral intention, and motivation—involving belief in organizational goals, willingness to exert effort, and desire to sustain membership. These form value commitment, effort commitment, and retention commitment, underpinning the widely used organizational commitment questionnaire (Y. Chen et al., 2019; Cheng et al., 2016; Fu, 2013; Mowday et al., 1979).

Within the broader field of organizational management research, organizational commitment has solidified its position as an indispensable factor. It serves multifaceted roles, functioning as a reliable indicator of employee effectiveness, a critical measure of employee attitudes, and a substantial determinant of overall organizational performance. Furthermore, it operates as a powerful predictive factor for employee retention or turnover behaviors (Meyer & Allen, 1997). Recent scholarship continues to affirm and extend these insights, particularly in high-stakes service industries like aviation. For example, among cabin crew, organizational commitment robustly predicts organizational citizenship behavior, with its effects moderated by high-performance human resource practices (Fu, 2013). In the context of the COVID-19 pandemic, it has also been identified as a vital mediator linking perceived organizational support to enhanced commitment and OCB, underscoring its resilience amid crisis-induced pressures (Alshaabani et al., 2021; Gupta, 2022).

### 2.3. Job Burnout

Edelwich (1980) identified burnout as a process comprising four stages: enthusiasm, stagnation, frustration, and apathy. Its causes include inadequate compensation, long working hours, career stagnation, lack of recognition, feelings of helplessness, and insufficient training, with interventions most effective during the initial enthusiasm stage. Shirom (1989) defined burnout as a persistent, negative, work-related state of mind characterized by exhaustion, reduced professional efficacy, and increased mental distance from one’s job. Drawing on conservation of resources theory, burnout encompasses three dimensions: physical fatigue, emotional exhaustion, and cognitive weariness. Demerouti et al. (2001) introduced the job demands-resources model, positing that workplace resources—physical, social, or organizational—mitigate job demands’ negative effects, enabling balance and goal attainment. Numerous studies applying this model demonstrate that high job demands elevate burnout and impair performance, while resources boost engagement and curb burnout (Bakker et al., 2004, 2014; C. Chen & Chen, 2012; Demerouti et al., 2001; Korunka et al., 2009; Lubbadeh, 2020).

Burnout adversely affects service quality, fostering depression, loss of enthusiasm, negative attitudes, diminished accomplishment, and indifference toward tasks and people. Its effects extend to organizational commitment, turnover intentions, and employee health (Kara, 2019; Lubbadeh, 2020). Cabin crew roles demand high emotional labor compared to other professions (C. Chen & Kao, 2011), spurring research on their burnout. For instance, Liang and Hsieh identified job satisfaction and career confidence as key burnout predictors among Taiwanese airline crew, with depersonalization linked to deviant behaviors and cynicism to performance declines. Chen and Kao applied the JD-R model to Taiwanese cabin crew, revealing burnout’s role in health issues and reduced performance amid demanding passengers and schedules. Heuven and Bakker (2003) examined emotional dissonance and burnout among German crew in a Dutch airline; Chen and Chen explored high-workload turnover intentions; and Mengenci showed burnout’s negative impact on job satisfaction among Turkish cabin crew and pilots (C. Chen & Chen, 2012; C. Chen & Kao, 2011).

### 2.4. Job Crafting

Wrzesniewski and Dutton (2001) defined job crafting as employees’ intentional changes to the task, relational, or cognitive boundaries of their job design to enhance personal satisfaction and meaning. They portrayed it as a bottom-up, employee-initiated process that aligns individual approaches with organizational objectives, yielding positive organizational outcomes. This construct manifests in three forms: (a) task crafting, such as altering tasks to pursue more engaging responsibilities; (b) relational crafting, by deepening ties with colleagues, supervisors, or clients; and (c) cognitive crafting, through reframing job perceptions to infuse greater purpose or mission.

Building on this, Tims et al. (2013) incorporated the job demands-resources model, conceptualizing job crafting as proactive adjustments to optimize the balance between job demands and resources. Their framework identifies three key dimensions: (a) increasing structural job resources (e.g., autonomy or support) to buffer demands; (b) seeking challenging demands to foster growth; and (c) reducing hindering demands (e.g., workload) to protect well-being. Petrou et al. (2012) echoed this with similar strategies: seeking resources, challenges, and demand reduction—such as negotiating autonomy or volunteering for developmental tasks. As a proactive behavior (Leana et al., 2009; Petrou et al., 2012), job crafting yields benefits like heightened organizational commitment (Ghitulescu, 2007; Iqbal, 2016; Leana et al., 2009; Romeo et al., 2019), organizational citizenship behavior (Ding et al., 2020; Shin & Hur, 2019), and reduced burnout (Chughtai & Rizvi, 2020; Singh & Singh, 2018; Tims et al., 2013). For instance, Cheng and O-Yang (2018) showed job crafting’s positive link to hospitality workers’ satisfaction, mediated negatively by burnout; Romeo et al. (2019) found high commitment mediating cognitive crafting’s effect on nursing care quality.

Recent cabin crew research underscores these dynamics. Karatepe and Eslamlou (2017) reported that job crafting boosts engagement, service recovery, and retention among crew, amplified by managerial support for task focus and confidence. Shin and Hur (2019) further demonstrated, via daily JD-R analysis, that flight attendants’ job crafting enhances OCB through job resources while curbing demands. Yet, COVID-19 restrictions have curtailed task crafting opportunities, limiting adaptive service innovations. Future studies should explore if crew can still harness job crafting to sustain motivation and commitment amid such constraints.

### 2.5. Perceived Organizational Support

Perceived organizational support is commonly defined as employees’ beliefs regarding the extent to which the organization values their contributions and cares about their well-being (Eisenberger et al., 1986). Key antecedents, including perceived fairness, supervisor support, rewards, and work conditions, significantly bolster POS (Eisenberger et al., 1986, 2001). In this study, Eisenberger et al. (1986) is therefore cited as the seminal work that originally conceptualized and operationalized perceived organizational support, while its contemporary relevance is substantiated through more recent empirical studies conducted in service and aviation settings, including during COVID-19 (e.g., Alshaabani et al., 2021; Gupta, 2022; Charoensukmongkol & Suthatorn, 2022). As a critical external resource, POS facilitates emotional recovery during task performance (Ji et al., 2019), which is especially vital for cabin crew amid their intense emotional demands.

Numerous studies, including recent ones amid COVID-19 (Alshaabani et al., 2021), show POS influences organizational commitment and job burnout (Gupta, 2022; Yaghoubi et al., 2014). Cabin crew research reveals high-performance work practices enhance POS, curbing turnover intentions while fully mediating links to counterproductive work behaviors (Fu, 2013). Among South Korean cabin crew, Kim et al. (2017) found POS exerts a direct positive effect on job performance, though it does not moderate emotional labor’s impact. Similarly, Charoensukmongkol and Suthatorn (2022) showed that Thai airlines’ vertical communication during the COVID-19 pandemic reduced insecurity for crew with high POS, but not for those with low POS. Therefore, organizational support plays a crucial role in mitigating the adverse effects of demanding work environments and fostering employee well-being (Nogues et al., 2021).

## 3. Method

### 3.1. Research Model and Hypotheses

**Figure 1:**
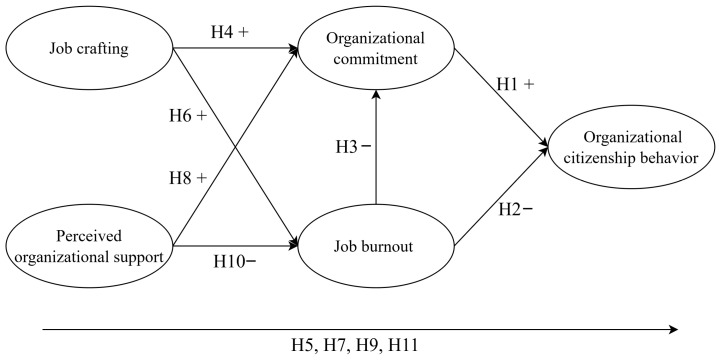
Research model.

Based on the theoretical discussion of relevant literature, this study posits that the influencing factors of organizational citizenship behavior among cabin crew include job crafting, perceived organizational support, job burnout, and organizational commitment. Accordingly, a comprehensive causal model is constructed. The research model framework is illustrated in Figure 1.

Organizational commitment refers to employees’ psychological attachment to the organization, motivating alignment with its goals and voluntary extra-role behaviors such as OCB (Grego-Planer, 2019; Kara, 2019). Empirical evidence confirms its positive effect on OCB (Alshaabani et al., 2021; Fu, 2013; Grego-Planer, 2019). For cabin crew, heightened commitment drives proactive actions like assisting colleagues and extra tasks, ensuring seamless operations through strong goal identification (Fu, 2013). Amid COVID-19-induced physical strain, stress, and emotional labor, organizational support bolsters commitment, enabling crew to deliver excellent service despite virus risks and quarantines (Alshaabani et al., 2021; Charoensukmongkol & Suthatorn, 2022). In this model, organizational commitment is conceptualized as an attitudinal construct (attachment and intention to remain), whereas OCB is conceptualized as a behavioral outcome (discretionary, organization-directed actions). Thus, commitment is expected to serve as a proximal attitudinal driver of OCB rather than overlapping with it conceptually. Thus, this study hypothesizes:

**H1.**  *Cabin crew members’ organizational commitment positively affects their organizational citizenship behavior.*

Burnout negatively impacts employees’ organizational citizenship behavior by impairing core duties and prosocial actions (Bateman & Organ, 1983; Maslach et al., 2001). For cabin crew, elevated burnout reduces passenger interactions to cope with stress (Maslach et al., 2001), job performance (Heuven & Bakker, 2003), and service quality (Mengenci, 2014), thereby curtailing OCB. This relationship merits re-examination amid post-pandemic challenges, motivating the present study. Thus, this study hypothesizes:

**H2.**  *Cabin crew members’ job burnout negatively affects organizational citizenship behavior.*

Previous research demonstrates that job burnout erodes organizational commitment (Kara, 2019; Maslach et al., 2001). Among cabin crew, burnout—stemming from work overload, irregular shifts, and emotional labor—diminishes psychological attachment, as seen in emotional dissonance effects (Heuven & Bakker, 2003). This link holds amid post-COVID stressors (Alshaabani et al., 2021), justifying re-examination. Thus, this study hypothesizes:

**H3.**  *Cabin crew members’ job burnout negatively affects organizational commitment.*

Previous research demonstrates that job crafting enhances organizational commitment by strengthening work identification, job satisfaction, and reducing turnover intentions (Ghitulescu, 2007; Iqbal, 2016; Romeo et al., 2019). For cabin crew, it boosts engagement, service performance, and retention amid demanding conditions (Karatepe & Eslamlou, 2017). Thus, this study hypothesizes:

**H4.**  *Cabin crew members’ job crafting positively affects organizational commitment.*

Job crafting enables cabin crew to integrate personal values into their roles, thereby enhancing organizational commitment (Iqbal, 2016; Romeo et al., 2019). This fosters stronger identification with organizational goals, prompting OCB such as assisting colleagues and extra-role efforts (Fu, 2013; Grego-Planer, 2019; Shin & Hur, 2019). Therefore, this study hypothesizes:

**H5.**  *Organizational commitment mediates the relationship between job crafting and organizational citizenship behavior of cabin crew members.*

Numerous studies demonstrate that job crafting reduces burnout (Chughtai & Rizvi, 2020; Singh & Singh, 2018; Tims et al., 2013). For cabin crew facing emotionally demanding roles, it enhances engagement, service recovery, performance, and retention (Karatepe & Eslamlou, 2017), while curbing job demands (Shin & Hur, 2019); restricted crafting exacerbates burnout, particularly amid post-pandemic constraints.

**H6.**  *Cabin crew members’ job crafting negatively affects job burnout.*

Previous studies show that job crafting positively affects OCB (Ding et al., 2020; Shin & Hur, 2019; Shusha, 2014). It also reduces burnout (Chughtai & Rizvi, 2020; Karatepe & Eslamlou, 2017; Singh & Singh, 2018; Tims et al., 2013), which in turn negatively influences OCB (Bateman & Organ, 1983; Maslach et al., 2001). Amid post-pandemic constraints like prevention policies that heighten burnout risks (Charoensukmongkol & Suthatorn, 2022), job crafting enables cabin crew to alleviate burnout, thereby enhancing OCB. Thus, this study hypothesizes:

**H7.**  *Cabin crew members’ job burnout mediates the relationship between job crafting and organizational citizenship behavior.*

Previous studies indicate that perceived organizational support positively influences organizational commitment (Alshaabani et al., 2021; Y. Chen et al., 2019; El-Aty & Deraz, 2018; Gupta, 2022; Rhoades et al., 2001). For cabin crew performing emotional labor, POS acts as a vital emotional recovery resource (Ji et al., 2019), fostering attachment through measures like layoff protections and staff reallocations during COVID-19 (Charoensukmongkol & Suthatorn, 2022; Dube et al., 2021). Post-pandemic, enhancing POS can alleviate work insecurity, curb turnover intentions, and strengthen commitment (Alshaabani et al., 2021; Charoensukmongkol & Suthatorn, 2022). Thus, this study hypothesizes:

**H8.**  *Cabin crew members’ perceived organizational support positively affects their organizational commitment.*

Organizational commitment mediates the positive relationship between perceived organizational support and organizational citizenship behavior (Alshaabani et al., 2021; Fu, 2013). Among cabin crew, POS fosters commitment, prompting proactive extra-role behaviors like assisting colleagues and handling extra duties (Fu, 2013). Post-COVID, support such as protective equipment and quarantine allowances enhances commitment amid virus risks, boosting OCB (Alshaabani et al., 2021; Charoensukmongkol & Suthatorn, 2022; Gupta, 2022). Thus, this study hypothesizes:

**H9.**  *Cabin crew members’ organizational commitment mediates the relationship between perceived organizational support and organizational citizenship behavior.*

Previous studies show that perceived organizational support reduces job burnout by buffering emotional exhaustion (Hur et al., 2013; Ji et al., 2019; Xu & Yang, 2021). For cabin crew, POS serves as a key emotional recovery resource amid demanding duties and post-pandemic stressors (Charoensukmongkol & Suthatorn, 2022; Ji et al., 2019). Thus, this study hypothesizes:

**H10.**  *Cabin crew members’ perceived organizational support negatively affects their job burnout.*

Previous studies demonstrate that perceived organizational support reduces job burnout by buffering emotional exhaustion (Hur et al., 2013; Ji et al., 2019; Xu & Yang, 2021, which in turn negatively affects organizational citizenship behavior (Bateman & Organ, 1983; Maslach et al., 2001). For cabin crew amid post-pandemic stressors, POS serves as a key emotional recovery resource (Charoensukmongkol & Suthatorn, 2022; Ji et al., 2019), alleviating burnout and thereby enhancing OCB. Thus, this study hypothesizes:

**H11.**  *Cabin crew members’ job burnout mediates the relationship between perceived organizational support and organizational citizenship behavior.*

### 3.2. Measurements

According to the research model, this study’s questionnaire measures five key variables: organizational citizenship behavior, job burnout, organizational commitment, job crafting, and perceived organizational support. All items were assessed using a 5-point Likert scale (1 = strongly disagree; 5 = strongly agree).

In terms of organizational citizenship behavior, this study refers to Buil et al. (2018) and Williams and Anderson (1991), defining cabin crew’s organizational citizenship behavior as voluntary efforts beyond formal job requirements to enhance organizational efficiency and benefit team development. Although the original plan considered the Williams and Anderson (1991)’s scale, K. Lee and Allen (2002) offers a refined version distinguishing organization-oriented (OCBO) from individual-oriented behaviors (OCBI) with 16 items total (Fu, 2013; Shin & Hur, 2019). However, Buil et al. (2018) selected three items from the OCBO scale, achieving high reliability (α = 0.770). To reduce respondent burden and prevent bias from uneven item distribution across dimensions, this study adopted Buil et al. (2018)’s version.

Regarding organizational commitment, this study employed the scale modified and validated by Alshaabani et al. (2021) and Charoensukmongkol and Suthatorn (2022). After tailoring the wording and dimensions to cabin crew’s work style, three sub-dimensions were identified: value commitment (two items, with their average score used), effort commitment, and retention commitment (Alshaabani et al., 2021; Charoensukmongkol & Suthatorn, 2022).

For job burnout, this study adopted the emotional exhaustion subscale from the Maslach Burnout Inventory developed by Maslach et al. (2001). Job burnout is defined as a syndrome involving prolonged mental, emotional, and physical exhaustion, including emotional exhaustion, cynicism (depersonalization), and reduced personal accomplishment (Maslach et al., 2001). Although prior cabin crew studies typically used the emotional exhaustion and cynicism subscales (C. Chen & Kao, 2011; Cheng & O-Yang, 2018; C. Lee et al., 2015; Liang & Hsieh, 2005), cynicism reflects long-term cumulative effects that are challenging to capture in this post-pandemic context (Charoensukmongkol & Suthatorn, 2022). Thus, emotional exhaustion—the core dimension—was measured using four items.

In terms of job crafting, it is defined by Wrzesniewski and Dutton (2001) as the changes individuals make in the tasks, relationships, or cognitive boundaries of their work to achieve greater satisfaction and fulfillment. Job crafting is generally categorized into task crafting, relational crafting, and cognitive crafting (Niessen et al., 2016; Shin & Hur, 2019; Tims et al., 2012; Tims & Bakker, 2010; Wrzesniewski & Dutton, 2001). This study developed a scale based on Niessen et al. (2016) encompassing these three sub-dimensions, with 9 items in total; the average scores of items within each sub-dimension were used for subsequent analysis.

For perceived organizational support, this study adopted the definition by Eisenberger et al. (1986), referring to employees’ beliefs about the extent to which the organization values their contributions and cares about their well-being. Most studies employ the scale developed by Eisenberger et al. (1986), as seen in Alshaabani et al. (2021), Appelbaum et al. (2018), Charoensukmongkol and Suthatorn (2022), Eisenberger et al. (2002), Thompson et al. (2020). In line with this tradition, our citation of Eisenberger et al. (1986) serves to acknowledge the original development of the POS construct and scale, whereas our theoretical arguments and hypotheses draw primarily on more recent POS research conducted in contemporary organizational and pandemic-related contexts. However, given the original scale’s numerous items suited mainly to corporate human resources contexts and cabin crew’s specific work environment, this study used the modified three-item scale from Eisenberger et al. (2002).

### 3.3. Data

This study developed the questionnaire items for the research variables by reviewing relevant literature from domestic and international scholars and adapting their established questionnaires. Following a pilot test with six cabin crew members, certain items were revised to enhance instrument quality. The target population consisted primarily of Taiwanese cabin crew members employed by the airline as of February 2023. As structural equation modeling was employed for data analysis, a minimum sample size of 200 was required. Due to constraints in time, budget, and personnel, the target sample size was set at 300 to avoid biases from excessively small or large samples, which could distort results or hinder data collection.

Cabin crew comprise multiple job levels; thus, quota sampling was used to ensure comprehensive representation across all ranks without bias toward any specific level. As of the end of 2021, C Airlines employed approximately 2933 cabin crew members. To ensure that different hierarchical ranks were proportionately represented, the population was divided into four rank groups and quota sampling was applied based on the official headcounts in each group. Specifically, the first group, ‘economy class crew (FY, MY)’, included about 1237 crew members (42.18% of all cabin crew), for which approximately 127 responses were targeted; the second group, ‘business class crew (FF, MF)’, included about 1272 crew members (43.37%), with a target of 131 responses; the third group, ‘pursers (PR) and junior pursers (JPR)’, included about 175 crew members (5.97%), with a target of 18 responses; and the fourth group, ‘cabin managers (CM)’, included about 249 crew members (8.49%), with a target of 26 responses. In total, the planned sample size was 302, allocated across quotas in proportion to the actual rank distribution.

To address any questions or uncertainties during completion, participants could contact the author via email or social media for clarification and assistance. This approach helped minimize missing responses and item misinterpretations, thereby enhancing data accuracy as the basis for the study’s analysis. A total of 369 valid questionnaires were collected, with no invalid ones. These included 49 males and 320 females, reflecting a higher female proportion consistent with the airline’s June 2022 crew gender ratio. The sample was predominantly aged 31–35 or 36–40 years, held university degrees, were married with children, and had 6–10 years of work experience—aligning overall with the airline’s cabin crew demographics.

## 4. Result

### 4.1. Reliability and Validity

This study assessed questionnaire reliability using Cronbach’s α coefficient. Higher α values indicate greater internal consistency among items measuring a construct. Values above 0.7 denote high reliability, 0.35–0.7 acceptable reliability, and below 0.35 low reliability (Cronbach, 1951). Results showed that Cronbach’s α for organizational citizenship behavior, organizational commitment, job burnout, job crafting, and perceived organizational support all exceeded 0.7, confirming good reliability across all constructs.

To verify the discriminant validity of the measurement model, this study compared the average variance extracted (AVE) for each construct against the squared correlations with other constructs, with AVE required to exceed these values. As shown in Table 1, most constructs met this criterion, except organizational citizenship behavior and organizational commitment. According to Hair (1998), discriminant validity is established if at least 75% of comparisons satisfy the condition, which applied here. For convergent validity, AVE should exceed 0.5 (values above 0.36 are marginally acceptable) (Fornell & Larcker, 1981); all constructs’ AVE values exceeded or approached 0.5, confirming convergent validity.

### 4.2. Confirmatory Factor Analysis

Confirmatory factor analysis verifies whether the survey data accurately measures the latent variables. The model in this study includes five latent variables—organizational citizenship behavior, organizational commitment, job burnout, job crafting, and perceived organizational support—each measured by 1 to 4 indicators. For the initial measurement model, $\chi^{2}$ = 209.006, *p* < 0.05, $\chi^{2}/df$ = 2.223 (within the acceptable range), GFI = 0.934, AGFI = 0.904, RMR = 0.048, SRMR = 0.0598, NFI = 0.930, TLI = 0.949, IFI = 0.960, and CFI = 0.960. With the exception of SRMR, which fell outside the “good” or “acceptable” range, all other fit indices were acceptable. This suggested issues among the latent variables in the initial model, necessitating adjustments. Examination of the path coefficients revealed that the path for the “relational crafting (V12)” indicator under job crafting was nonsignificant and thus deleted. Following this modification, model fit improved: GFI increased to 0.951, AGFI to 0.926, RMR decreased to 0.040, SRMR to 0.0452, RMSEA to 0.046, NFI to 0.951, TLI to 0.971, IFI to 0.978, and CFI to 0.978, indicating good overall fit.

Subsequently, measurement model characteristics were analyzed, with results presented in Table 2. As shown, all standardized factor loadings were statistically significant and exceeded 0.5. Moreover, composite reliability values for each construct surpassed 0.6, demonstrating good consistency among the indicators and meeting the standards proposed by Fornell and Larcker (1981). Overall, the revised measurement model exhibited strong construct validity.

### 4.3. Path Analysis

This study employed path analysis on the proposed model to examine causal relationships. The overall fit indices were $\chi^{2}$ = 148.590, *p* < 0.05, $\chi^{2}/df$ = 1.812, GFI = 0.949, AGFI = 0.926, RMR = 0.042, SRMR = 0.0464, RMSEA = 0.047, NFI = 0.949, TLI = 0.970, IFI = 0.976, and CFI = 0.976, indicating acceptable model fit. Four paths showed significant t-values, supporting four hypotheses. Results are presented in Table 3.

Firstly, H10 (Cabin crew organizational commitment has a positive impact on organizational citizenship behavior) yielded a standardized path coefficient of 0.846, supporting H1. Thus, as organizational commitment increases, so does organizational citizenship behavior, confirming H1. In other words, when cabin crew members desire to remain with the company, are willing to exert extra effort, feel emotionally attached to the organization, and accept additional tasks, they are more likely to voluntarily engage in behaviors beneficial to the organization, such as participating in non-mandatory activities that enhance the company’s image and protecting it from potential risks. Although the standardized path coefficient between organizational commitment and organizational citizenship behavior (OCB) is high, the two constructs are clearly distinct in both theory and measurement. Organizational commitment is defined as employees’ psychological attachment to the organization that develops through compliance, identification, and internalization, aligning employees with organizational goals and motivating extra effort. By contrast, OCB refers to discretionary, voluntary behaviors beyond formal job requirements that are not explicitly prescribed or rewarded by official compensation systems. This study also separates the two: organizational commitment is assessed through value, effort, and retention commitment, whereas OCB is captured with items focusing on organization-directed voluntary behaviors that enhance efficiency and team development. Accordingly, organizational commitment is treated as an attitudinal attachment antecedent, whereas OCB is modeled as a behavioral, discretionary outcome; their strong correlation reflects an attitude–behavior link rather than conceptual overlap.

Secondly, “H4: Cabin crew job crafting has a positive impact on organizational commitment” showed a standardized path coefficient of 0.740 (t = 6.237), supporting H4. This indicates that job crafting by cabin crew members significantly and positively affects their organizational commitment. When they adjust their work to better suit their needs and derive personal meaning from it, they are more inclined to feel emotionally committed and willing to invest extra effort.

Furthermore, “H8: Cabin crew perceived organizational support has a positive impact on organizational commitment” had a standardized path coefficient of 0.175 (t = 2.062), supporting H8. When cabin crew members perceive that the airline values their goals, contributions, and well-being while providing good benefits, their organizational commitment strengthens, increasing their willingness to contribute more, accept assignments, remain with the organization, engage diligently in work, support the company during the pandemic, and comply with health regulations.

Conversely, “H10: Cabin crew perceived organizational support has a negative impact on job burnout” produced a standardized path coefficient of −0.267 (t = 3.235), supporting H10. When cabin crew members feel valued in terms of goals, contributions, and welfare, their perceived organizational support rises, thereby reducing emotional exhaustion and mental strain associated with job burnout, especially amid pandemic prevention measures.

However, three hypotheses were not supported: “H2: Cabin crew job burnout has a negative impact on organizational citizenship behavior,” “H3: Cabin crew job burnout has a negative impact on organizational commitment,” and “H6: Cabin crew job crafting has a negative impact on job burnout.” Job burnout did not significantly affect organizational citizenship behavior or commitment (path coefficients: 0.064 and −0.064, respectively). This suggests that, amid the pandemic, cabin crew members’ job burnout had no discernible influence on these outcomes. Similar findings have been reported in prior studies, possibly due to intervening factors or a nonlinear relationship—for instance, low or high burnout levels may have minimal effects, while moderate levels could even be positive. Future research should explore this further.

Additionally, the effect of job crafting on job burnout was nonsignificant, likely because external changes prevented cabin crew members from translating job crafting into reduced burnout. Notably, all three unsupported hypotheses involved job burnout, and the mean scores for burnout items ranged from 2.84 to 3.24, reflecting relatively low levels with limited variation. This may stem from pandemic-related reductions in workdays, lighter workloads, and infrequent scheduling (less than once a month), resulting in low emotional exhaustion. Such conditions likely attenuated the relationships between job burnout and other variables.

### 4.4. Mediating Effect Analysis

In this study’s theoretical model, organizational commitment and job burnout were originally posited as mediating variables. However, since Hypothesis H2 (“Cabin crew job burnout negatively affects organizational citizenship behavior”) was not supported, Baron and Kenny (1986)’s four-step method indicates that job burnout does not exhibit a mediating effect. Consequently, Hypotheses H7 and H11 were not supported, and mediating effect analysis was conducted solely for organizational commitment. This study employed the bootstrap method for testing, which treats the sample itself as the population and conducts repeated resampling. The estimated values from these 2000 iterations generate a new distribution, enabling calculation of the confidence interval. If the confidence interval excludes zero, the effect is statistically significant. As shown in Table 4, the confidence intervals for the mediating effect of organizational commitment—in the paths from job crafting and perceived organizational support to organizational citizenship behavior—do not include zero, confirming its statistical significance.

Furthermore, this study examined the mediating role of organizational commitment using Baron and Kenny (1986)’s four-step method. As shown in the prior path analysis results, both job crafting and perceived organizational support significantly influence organizational commitment, which in turn significantly affects organizational citizenship behavior. Thus, it sufficed to verify the direct effects of job crafting and perceived organizational support on organizational citizenship behavior, followed by an assessment of these effects after incorporating organizational commitment, to determine whether complete or partial mediation was present.

The results indicated significant direct effects of job crafting (path coefficient = 0.446) and perceived organizational support (path coefficient = 0.306) on organizational citizenship behavior. However, upon including organizational commitment in the model, these effects became nonsignificant (path coefficients = −0.367 and 0.128, respectively). These findings demonstrate that organizational commitment fully mediates the effects of job crafting and perceived organizational support on organizational citizenship behavior, indicating complete mediation.

## 5. Discussion

### 5.1. Managerial Implications

This study derives the following managerial implications from its key findings. First, since organizational commitment positively influences organizational citizenship behavior (H1 supported), airlines should implement targeted human resource strategies to foster stronger commitment, thereby elevating OCB. These include providing career development programs, specialized training opportunities, performance-based incentives, and supportive work environments. Additionally, prioritizing employee well-being through psychological counseling services, comprehensive health insurance, and stress management initiatives can mitigate anxiety, enhance physical and mental health, and ultimately boost work engagement, loyalty, and voluntary contributions that drive organizational growth.

Second, job crafting positively affects organizational commitment (H4 supported). To amplify OCB indirectly via heightened commitment, airlines should encourage job crafting among cabin crew. For general crew members, this entails allowing them to select preferred tasks during flights, promoting task customization that aligns with personal strengths and increases policy adherence and loyalty. For cabin managers, granting flexibility in leadership styles and interpersonal approaches—within established guidelines—can outperform rigid protocols, enabling more personalized passenger interactions. Thus, airlines should avoid inflexible duty rosters and rigid shift schedules to enhance autonomy and job satisfaction.

Third, perceived organizational support positively influences organizational commitment (H8 supported) while negatively impacting job burnout (H10 supported). During the pandemic, flight reductions undermined job security, eroding POS and commitment. As recovery progresses with increased hiring, retaining talent demands bolstering POS through optimized workspaces, emotional support resources, streamlined procedures, stringent yet supportive pandemic protocols, enhanced benefits, and regulatory flexibility. These measures empower crew to feel valued, fostering creativity, choice, and sustained engagement.

Notably, job burnout showed no significant effects on organizational commitment (H3) or OCB (H2), nor was it significantly reduced by job crafting (H6). This may reflect moderating influences such as job satisfaction, personal values, or job characteristics (Halbesleben, 2010). Pandemic-induced job insecurity might have prioritized retention, maintaining commitment and OCB despite burnout, while reduced workloads from fewer flights lowered exhaustion levels without proportionally elevating commitment, as observed in related studies. Under these unique conditions, airlines should tailor burnout interventions contextually, monitoring evolving post-pandemic dynamics. In interpreting the non-significant findings for H2, H3, and H6, it is important to note that burnout in this study was operationalized only through emotional exhaustion. While emotional exhaustion is considered the core component of burnout, cynicism and reduced personal accomplishment capture more chronic attitudinal and evaluative aspects of the syndrome that may be more proximal to changes in organizational commitment and OCB. By excluding these dimensions, the present measurement may have underestimated the overall level and variability of burnout, thereby attenuating its statistical associations with other constructs.

### 5.2. Suggestion for Future Research

This study offers the following recommendations for future research. First, limited to Taiwanese cabin crew from one airline, this study lacks generalizability, particularly in nationality diversity. Future studies should sample cabin crew from diverse nationalities and airlines to enhance representation and enable comparative analyses of organizational citizenship behavior, organizational commitment, work burnout, job crafting, and perceived organizational support.

Second, a major limitation of this study is that job burnout was measured only by the emotional exhaustion subscale of the Maslach Burnout Inventory, whereas cynicism and reduced personal accomplishment were omitted. Although this choice was motivated by concerns about capturing cumulative effects in a post-pandemic context, it restricts the construct validity and depth of our burnout analysis. It is possible that the absence of these additional dimensions contributed to the non-significant paths for H2, H3, and H6, as our burnout indicator may not fully reflect more enduring detachment and diminished efficacy that are theoretically linked to organizational commitment and OCB. Future research should employ the full burnout scale to re-examine these relationships.

Third, this study faces an important contextual limitation related to the relatively low and compressed levels of emotional exhaustion observed in our sample and the specific timing of data collection. By February 2023, participants were exclusively cabin crew who had remained with the airline throughout the pandemic and its initial recovery, meaning that those who had experienced the most severe burnout may already have resigned or transferred. This potential survivor bias likely resulted in a more resilient group whose organizational citizenship behavior and commitment were less sensitive to burnout. At the same time, data collection coincided with a phase of gradual industry recovery, during which route resumptions, improving job security, and renewed career prospects may have temporarily boosted motivation and willingness to “go the extra mile,” even among employees reporting moderate exhaustion. Together, these sample-selection and timing effects may have contributed to the low variance in burnout scores and, consequently, to the non-significant relationships involving burnout (H2, H3, and H6). Future longitudinal studies that span different stages of industry recovery and, where feasible, also include employees who have left the organization are needed to more rigorously examine these dynamics.

Furthermore, post-pandemic psychological shifts in cabin crew remain under-explored, despite evidence that COVID-19 fundamentally altered their work conditions, emotional labor, and exposure to health-related risks. The absence of pre-pandemic baseline data constrains the ability to capture trajectories of burnout, commitment, perceived organizational support, and OCB over time, and limits causal inferences about how crisis-related changes in job demands and resources shape these constructs. Future studies should therefore employ longitudinal panel designs that follow cabin crew across different phases of industry recovery (e.g., initial rebound, network expansion, and normalization), in order to map psychological adjustment processes and identify delayed or cumulative effects. In addition, integrating qualitative approaches—such as in-depth or narrative interviews and focus groups—would allow researchers to uncover nuanced changes in meaning-making, identity, and job crafting strategies that may not be fully captured by standardized scales, thereby deepening theoretical understanding and enhancing the rigor and ecological validity of post-pandemic research on cabin crew.

Additionally, focusing on retained crew from an organizational perspective, this study adopted the conservation of resources (COR) framework to examine job crafting and perceived organizational support as key resource-based antecedents of burnout, but, due to time and questionnaire-length constraints, it did not explicitly incorporate job demands from the job demands–resources (JD-R) model, such as workload, emotional labor intensity, schedule irregularity, or role conflict. This resource-centered approach is informative for understanding how crew members protect and build valued resources through job crafting and POS, but it provides only a partial view of the JD-R process in which demands and resources jointly shape strain and motivation. As pandemic-related pressures and operational disruptions gradually subside, future research should more fully operationalize JD-R job demands—capturing, for example, emotional dissonance, demanding passengers, long or irregular duty periods, and safety responsibilities—to investigate how specific demand profiles interact with job crafting and POS to predict burnout, organizational commitment, and organizational citizenship behavior among both retained and newly hired crew.

Finally, the findings should be interpreted in light of the specific Taiwanese cultural and organizational context. Taiwanese society is often characterized as more collectivistic and higher in power distance, with strong norms of harmony and loyalty, which may strengthen the links between perceived organizational support, commitment, and OCB among cabin crew compared with more individualistic cultures. In addition, the focal carrier is a traditional full-service airline with relatively stable employment relations and stronger emphasis on safety and service quality than on cost-cutting, which may partly explain the relatively low burnout levels and the strong mediating role of organizational commitment. Future research could therefore conduct cross-cultural and cross-model comparisons, for instance between Taiwanese and non-Taiwanese crews and between full-service and low-cost carriers, to examine whether the structural relationships among POS, job crafting, burnout, commitment, and OCB hold or differ across cultural and organizational settings.

## 6. Conclusions

The primary objective of this study is to examine whether cabin crew members’ organizational citizenship behavior in the post-pandemic era is influenced by their perceived organizational support and job crafting, with work burnout and organizational commitment as mediating variables. The results show that when cabin crew members wish to continue their employment with the company, are willing to make extra efforts, and feel emotionally committed to the organization, they tend to engage in more voluntary behaviors benefiting the organization. In other words, as organizational commitment among cabin crew members increases, their organizational citizenship behavior also improves. These findings are consistent with previous studies (Alshaabani et al., 2021; Fu, 2013; Grego-Planer, 2019), supporting hypothesis H1. Second, job crafting positively influences organizational commitment among cabin crew members, as evidenced by significant path analysis results. This effect holds regardless of whether the crew member holds a managerial or senior position, aligning with previous studies (Ghitulescu, 2007; Iqbal, 2016; Leana et al., 2009; Romeo et al., 2019) and supporting hypothesis H4. Furthermore, perceived organizational support significantly and positively affects organizational commitment among cabin crew members, corroborating previous research and supporting hypothesis H8. Finally, perceived organizational support significantly and negatively impacts work burnout among cabin crew members, consistent with previous studies and supporting hypothesis H10. Conversely, work burnout was found to negatively affects cabin crew members’ organizational citizenship behavior, but the effect was not statistically significant, failing to support hypothesis H2. This result aligns with prior findings in the literature. Similarly, the negative path from work burnout to organizational commitment was not significant, failing to support hypothesis H3, consistent with Ko. Since job crafting did not significantly affect work burnout, hypothesis H6 was also not supported. Moreover, this study confirms that organizational commitment fully mediates the relationship between job crafting and organizational citizenship behavior, supporting hypotheses H5 and H9. However, as work burnout does not significantly impact organizational citizenship behavior, no mediating effect exists, and hypotheses H7 and H11 were not supported.

## Figures and Tables

**Table 1:** Discriminant validity.

Organizational citizenship behavior	0.484				
Organizational commitment	0.704	0.618			
Job burnout	0.015	0.051	0.612		
Job crafting	0.432	0.762	0.023	0.479	
Perceived organizational support	0.342	0.423	0.067	0.398	0.746

Note: The diagonal represents the AVE values, while the off-diagonal elements represent the squared values of the correlation coefficients.

**Table 2:** Analysis of the revised measurement model.

	Standardized Factor Loading	*t*-Value	Composite Reliability
Organizational citizenship behavior			0.737
V1. participate in voluntary activities that are beneficial to the company’s image.	0.729	14.579 *	
V2. I am willing to offer suggestions for matters that are beneficial to the company’s operations.	0.653	12.726 *	
V3. When the company faces potential problems or risks, I am willing to take action to protect the company.	0.703	13.924 *	
Organizational commitment			0.829
V4. Value commitment: V4-1. In order to remain in the company, I am willing to accept any tasks assigned to me by the company. V4-2. I feel proud when I talk to others about my work in this company.	0.736	15.631 *	
V5. Effort commitment: I am willing to put in extra effort to help the company achieve greater success.	0.817	18.046 *	
V6. Retention commitment: I have a strong emotional connection with this company.	0.722	15.225 *	
Job burnout			0.862
V7. The epidemic prevention policies during the pandemic made me feel mentally drained from work.	0.639	12.989 *	
V8. After a whole day of work, I feel exhausted.	0.829	18.486 *	
V9. Every morning, thinking about facing another day of work makes me feel tired.	0.859	19.460 *	
V10. A full day’s work leaves me feeling mentally strained.	0.783	17.064 *	
Job crafting			0.637
V11. Task crafting: V11-1. I focus on specific tasks and duties that make my work more suitable for me. V11-2. I take on or seek additional tasks and duties that make my work more suitable for me. V11-3. I focus more on tasks and duties that I enjoy.	0.529	9.879 *	
V13. Cognitive crafting: V13-1. I try to view my work as having deeper meaning. V13-2. I find personal meaning in my work. V13-3. I believe that part of my work is more than just tasks and responsibilities.	0.824	14.860 *	
Perceived organizational support			0.896
V14. The company greatly values my contributions to it.	0.955	24.017 *	
V15. The company highly values my goals and values.	0.938	23.306 *	
V16. The company greatly values my well-being.	0.667	14.215 *	

* indicates that the *t*-test reached a significant level (*p*-value < 0.05).

**Table 3:** Path analysis of the research model.

	Standardized Path Coefficients	*t*-Value	R^2^
Organizational citizenship behavior			0.696
Organizational commitment (H1)	0.846	11.750 *	
Job burnout (H2)	0.064	1.234	
Organizational commitment			0.768
Job burnout (H3)	−0.064	−1.332	
Job crafting (H4)	0.740	6.237 *	
Perceived organizational support (H8)	0.175	2.062 *	
Job burnout			0.066
Job crafting (H6)	0.015	0.162	
Perceived organizational support (H10)	−0.267	−3.235 *	

* indicates that the *t*-test reached a significant level (*p*-value < 0.05).

**Table 4:** Result of mediating effect analysis.

	95% Confidence Interval
Job crafting → Organizational commitment → Organizational citizenship behavior (H5)	[0.626, 0.829]
Perceived organizational support → Organizational commitment → Organizational citizenship behavior (H9)	[0.397, 0.659]

## Data Availability

The data presented in this study are available upon request from the corresponding author.

## Abbreviations

The following abbreviations are used in this manuscript:

COVID-19	Coronavirus Disease 2019
OCB	Organizational Citizenship Behavior
POS	Perceived organizational support
OCBO	Organizational Citizenship Behavior—Organization
OCBI	Organizational Citizenship Behavior—Individual
AVE	Average Variance Extracted
